# Human Trafficking: Results of a 5-Year Theory-Based Evaluation of Interventions to Prevent Trafficking of Women From South Asia

**DOI:** 10.3389/fpubh.2021.645059

**Published:** 2021-05-17

**Authors:** Cathy Zimmerman, Joelle Mak, Nicola S. Pocock, Ligia Kiss

**Affiliations:** ^1^Gender Violence & Health Centre, Department of Global Health & Development, London School of Hygiene & Tropical Medicine, London, United Kingdom; ^2^Lumos Foundation, London, United Kingdom; ^3^Faculty of Population Health Science, Institute for Global Health, University College London, London, United Kingdom

**Keywords:** human trafficking, modern slavery, migrant women, realist evaluation, South Asia

## Abstract

Preventing modern slavery is of global interest, but evidence on interventions remains weak. This paper presents findings from a 5-year theory-based evaluation of an empowerment and knowledge-building intervention to prevent the exploitation of South Asian female migrant workers. The evaluation used realist evaluation techniques to examine the intervention mechanisms, outcomes, and context. Findings from qualitative and quantitative data from Nepal, India, and Bangladesh indicate that the intervention mechanisms (trainings) were not well-targeted, not delivered by appropriate trainers, and did not address participants' expectations or concerns. The outcomes of empowerment and migration knowledge were not achieved due to poor integration of context-related factors, flawed assumptions about the power inequalities, including barriers preventing women from asserting their rights. Ultimately, interventions to prevent exploitation of migrant workers should be developed based on strong evidence about the social, political, and economic realities of their migration context, especially in destination settings.

## Introduction

Human trafficking is a global phenomenon that touches most corners of the world, with ~40.3 million individuals estimated to be in situations of forced labor and forced marriage—broadly referred to as “modern slavery” (1). Studies over the past two decades have increasingly documented the physical, psychological, and socioeconomic harm caused by extreme exploitation, which generally affects the world's most vulnerable adults and children (2, 3). Yet, there is astonishingly limited evidence on “what works” to prevent these abuses or to address the consequences (4). In a recent systematic review including 90 reports on trafficking programs, the authors concluded that organizations are still “struggling to demonstrate impact and discern *what works* to combat human trafficking” (5). Similarly, a Rapid Evidence Review of interventions in South Asia noted that “…the outcomes from the reviewed studies alone cannot be used as recommendations for policy and practice on trafficking…” (6). These findings undoubtedly come as disheartening news to the many policy-makers and donors who seek evidence-informed avenues to meet the 2030 Sustainable Development Goal Target 8.7, which aims to eradicate forced labor, end modern slavery and human trafficking, and secure the prohibition and elimination of the worst forms of child labor.

### Human Trafficking in Nepal, India, and Bangladesh

The Asia and the Pacific region account for over half of the ~24.9 million people in forced labor globally. India is estimated to have the largest number of persons in modern slavery globally at 8 million, compared with an estimated 592,000 in Bangladesh and 171,000 in Nepal (7). Among those in situations of forced labor, the largest prevalence, 24%, are estimated to be in domestic work (1), where exploitation by employers and labor intermediaries are common.

### Awareness and Knowledge-Building Antitrafficking Interventions

Premigration community-based awareness and knowledge building activities have been particularly popular among implementing agencies and donors because they are of relatively low risk and can reach large populations at fairly low cost (8). Safe-migration interventions are generally based on the assumption that if people had better knowledge about migration-related risks and regulations and knew their rights, they could avoid trafficking-related abuses and migrate safely (9). However, to date, there is no robust evidence that premigration knowledge promotes safer migration (9–12). Of activities that have been assessed, to whatever extent, the vast majority of evaluations has only measured outputs (e.g., number of sessions and participants) and intermediate outcomes (e.g., immediate levels of knowledge and awareness). No evaluations have measured how increased awareness or knowledge affects individual behaviors and, in turn, how different behaviors might affect incidence or prevalence of human trafficking or modern slavery (13). Studies examining awareness-raising and knowledge-building activities, including those using experimental designs, have stopped at the point of assessing whether messages were learned by the participants vs. tracking how the new knowledge was applied or measuring how it affected migration-related safety or trafficking outcomes (10, 14, 15). To date, there are no data indicating modifiable factors or actionable targets to reduce “vulnerability” to trafficking. Studies attempting to identify determinants have cited primarily broad development problems, such as “low income; few economic opportunities, and instability of the local economy” (16) or ‘'poverty,” “gender,” and “age” (9). Unfortunately, these insights are not particularly helpful for anti-trafficking programming because they could relate to most ailments in low-resource settings, which development programmes have been trying to address for decades. Moreover, for intervention purposes, these broad determinants are not generally actionable within current anti-trafficking budgets or timeframes.

### SWIFT Study of the Work in Freedom Program in South Asia

The South Asia Work in Freedom Transnational Evaluation (SWIFT) was a 5-year program of research and evaluation in Nepal, India, and Bangladesh of the International Labor Organisation's (ILO) Work in Freedom Programme (WIF) (17). The SWIFT evaluation was conducted independently and sought to answer the question: *How do the WIF community-based prevention interventions influence women's risk of forced labor (modern slavery) in domestic work and garment sectors?* SWIFT is the first evaluation to follow a large-scale, multicountry trafficking intervention, from conception to implementation, using theory-based mixed-methods approaches. The WIF program theory was based on the concept that trafficking could be prevented by predeparture community activities comprised of women's empowerment strategies, including training on the value of women's work, the costs and benefits of migration, safe and informed migration, women's rights and workers' rights and knowledge, and skills capacity building (18). Sessions in some sites also included components to improve women's ability to make informed livelihood decisions either by equipping them to migrate safely or access to local livelihood options if they did not want to migrate. This paper analyses the combined SWIFT theory-based evaluation findings across the three country intervention settings, namely, India, Nepal, and Bangladesh, and examines the intervention theory, context, mechanisms, and outcomes. WIF in India focused on internal migration, while in Nepal and Bangladesh, the target was international migration.

## Theory and Methods

**Figure 1:**
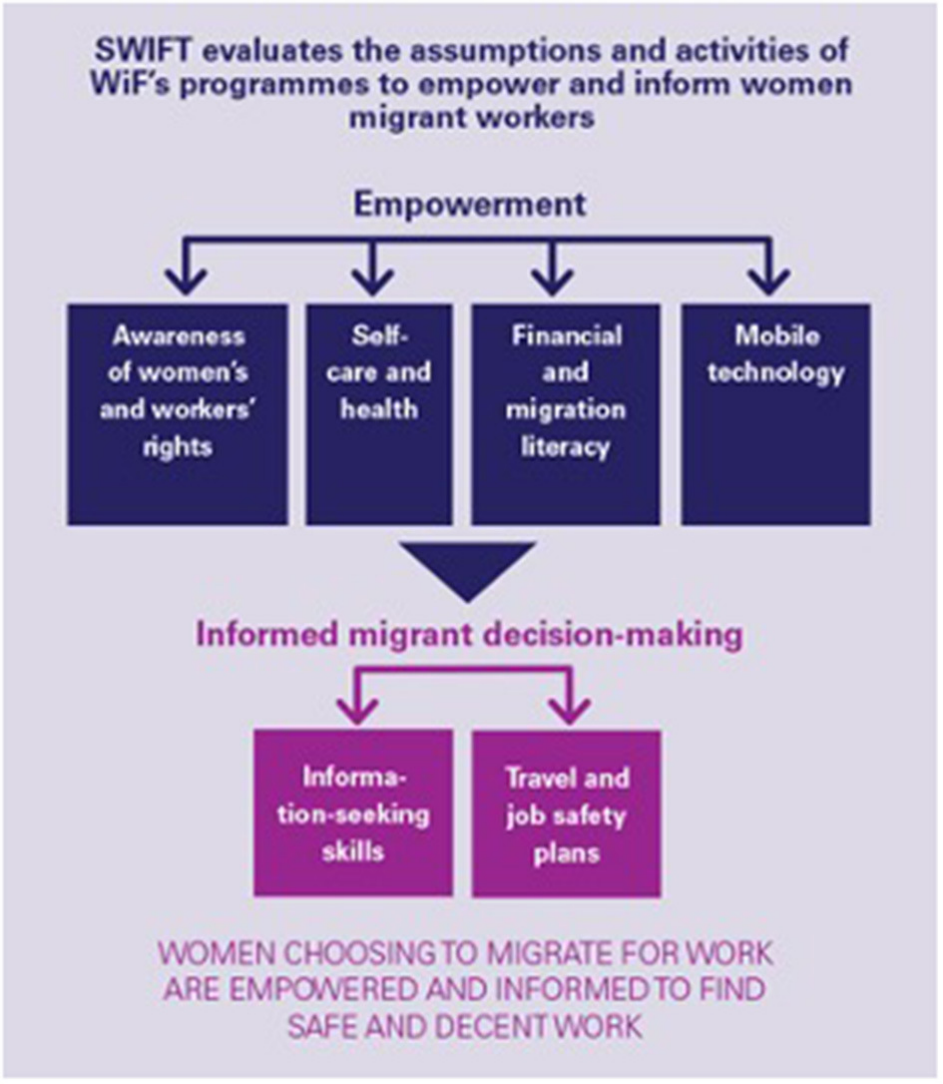
Work in Freedom (WIF) theory, assumptions, and activities evaluated using theory-based methods.

SWIFT applied a mixed methods theory-based approach to evaluate the theory and implementation of the WIF program. Specifically, SWIFT investigated the validity of the intervention's theory and underlying assumptions that labor trafficking could be reduced or eliminated by enhancing women's autonomy and generating adoption of safe migration practices (Figure 1). SWIFT examined whether empirical evidence supported the theory that migrant women's greater awareness and knowledge would decrease their risk of exploitation and explored implementation, causal pathways, and detectable effects on knowledge transfer, uptake, and application. Findings were intended to inform future replication and scale-up.

A theory-based realist evaluation design was selected because of the early stage of intervention development and the programmatic need for evidence on intervention designs and implementation. Several important considerations influenced the evaluation design. First, the space–time complexity of human trafficking demands research approaches that integrate causal thinking to capture the complex causes–effects interactions vs. methods that tend to isolate single causes of observed effects (19, 20). Second, many core components of the intervention were still under development, and the main outcome was relatively undefined at the start of WIF implementation. Third, at the start of WIF, there was little evidence on risks and protective factors to support the hypothesized causal pathways from *empowerment* to *protection against exploitation*, which meant that focusing on effectiveness measurement would not provide useful results (21). That is, even if our evaluation indicated some level of intervention effectiveness, we would not know if it could work elsewhere (22). If the evaluation did not find positive results, we would not know whether there was a problem with the implementation or with the theory. Fourth, the intervention's feasibility, acceptability, and uptake had not been previously assessed to justify a complex and costly experimental design, making it very difficult to assess external validity. Fifth, collection of SWIFT monitoring data specifically for the evaluation was not possible due to local constraints. Consequently, we could not conduct a process-outcome evaluation, which could have allowed us to more rigorously investigate processes of change, assess quality of implementation, and identify contextual factors linked to variations in outcomes (23).

Thus, to capture the subject's complexity, the nascent stage of the intervention, and to achieve the explanatory power needed, we used a multisite mixed-methods design and adopted innovative analytical approaches, which served as strong tools for reasoning under uncertainty (24). Following realist principles, we did not expect to find a definitive answer to the question of *what works* to prevent human trafficking but rather aimed to investigate what mechanisms were relevant to preventing human trafficking in the intervention contexts (25). The intended “impact,” women who choose to migrate for work are “empowered and informed to find safe and decent employment,” was the *ceiling of accountability*^1^ for the WIF program vs. “prevention of trafficking” (see Figure 1). Using data from the three countries, we examined constraints and enablers for the immediate training outcomes (empowerment and information) and to the ultimate intended impact (reduced trafficking).

### Forced Labor Measurement

To assess prevalence and types of exploitation within our study population, we followed the ILO's guidance by assessing three dimensions of forced labor: (1) unfree recruitment, (2) work and life under duress, and (3) impossibility of leaving the employer (27). For each dimension, a range of involuntariness and penalty indicators were measured, and these were classified as medium or strong, according to the ILO guidelines. Indicators were constructed from a number of questions asked in the survey about specific individual experiences (28). Participants who reported at least one indicator of involuntariness and one penalty within a dimension, of which one was a strong indicator, were defined as having experienced that dimension of forced labor. Experience of any one of the three dimensions constitutes forced labor (27).

### Study Methods for Each Study Site

In Nepal, formative research was conducted in collaboration with the Centre for the Study of Labour and Mobility, Social Science Baha (CESLAM) in the district of Dolakha prior to the rollout of the community component of the WIF intervention. The formative research was conducted to provide initial prevalence figures on migration, as such data were not available. Subsequent research was conducted in three of the five WIF districts, Chitwan, Rupandehi, and Morang and included 519 returnee migrants who had previously migrated internationally for work purposes and 340 prospective migrants who planned to migrate internationally for work. Qualitative semistructured interviews were also carried out with 55 of the prospective migrant women who participated in the survey, of which six were reinterviewed following their attendance of the WiF 2-day training. Participants interviewed for the prospective migrant survey were followed up by telephone to track their migration process, including after their departure from Nepal. At the first follow-up, 188 women were reached, representing an attrition rate of 45%. Of the 188 women, one-third reported that they were no longer planning to migrate, reducing the sample size to 130. At the second follow-up, only 10 women participated, and the data were not included in further analysis.

In India, surveys were conducted in collaboration with the Centre for Women's Development Studies (CWDS) with 4,671 households, including detailed survey interviews with 1,218 women and 1,156 men in 20 probabilistically selected villages across the Ganjam District of Odisha. Among the participants, 112 women and 429 men had previously migrated (29). Pre- and posttraining questionnaires were also administered with women (*N* = 347) who participated in WIF's 2-day premigration training session. To gage awareness levels, participants were asked a single question relevant to a concept and asked to answer free form in their own words, i.e., interviewers did not read out item lists. For example, to examine awareness of the benefits of migrating for work, women were asked: “What would you say are the main benefits of moving away from home to take up work somewhere else?” Enumerators could then select any of nine benefits that women could name (30). Vignettes were also used in some questions prior to eliciting women's awareness of particular concepts. Such depersonalization encouraged participants to reflect beyond their own individual circumstances, which is particularly useful when discussing sensitive topics (31).

In Bangladesh, qualitative research was carried out in collaboration with Drishti Research Centre, in three research sites in the district of Narayanganj: (i) a rural area; (ii) a former resettlement area for Dhaka slum dwellers—now an industrial district; and (iii) a densely populated semiurban area. The team conducted ethnographic observations and interviewed a cohort of 40 migrant women who participated in the WIF training and stated intentions to migrate. Women were interviewed five times between December 2015 and May 2017 (32).

In the intervention sites in Nepal and Bangladesh, most women who migrated internationally were engaged in domestic work. In the India intervention sites, most women migrated internally and the largest portion worked in construction.

### Analysis

Analysis for each research component was conducted and reported separately by country. In this paper, we conducted a synthesis of findings across the different studies, based on principles of realist evaluation. We consider the implications for future prevention interventions designed to inform and empower women to find safe and decent work and, in turn, avoid abuse and exploitation at destination.

Using the formative data collected in Nepal, we estimated key characteristics of migrant households and returnee migrants, including prevalence of labor migration, and findings on remittances, mobile phone use, common work sectors, and reported injuries (33). In the other three sites, we examined migration planning among prospective Nepali migrant women by comparing first-time and repeat migration using logistic regressions controlling for district, time until proposed departure, and age. Further methodological detail is available elsewhere (34).

To analyze past labor experiences and remigration intentions among Nepali returnee women, we examined their remigration intentions against their experiences (or not) of forced labor during their most recent migration. We used Bayesian networks to model the Nepal returnee dataset to examine causal interactions between variables and make predictions about the effect of potential interventions. We used multivariate logistic regression to examine the association between forced labor experience and speaking the destination's language among women who migrated to Arabic-speaking countries. The model was adjusted for destination country and work sector, based on the conceptual framework and findings published in the article by Kiss et al. (20).

Thematic analysis was used to analyze semistructured interviews in Nepal, and discourse and thematic analysis were used to analyze interviews in Bangladesh (32, 35).

Descriptive analysis was used to describe the context of WIF's implementation in Odisha. We calculated the number of migrant households, prevalence of migration, predeparture indicators, working conditions, and prevalence of forced labor among migrants by gender. To analyze the pre- and posttraining data, we used descriptive analysis and unadjusted analyses (paired *t*-tests, McNemar's tests, Wilcoxon signed ranks tests) to estimate the differences in scores before and after predeparture awareness training. Adjusted analyses used mixed effects models to explore whether receiving information on workers' rights or working away from home prior to the training was associated with changes in before-and-after scores. Further information on analysis is available elsewhere (30).

## Results

We report overall findings using the Context–Mechanism–Outcome framework in realist evaluation (36). We first describe findings by Mechanisms of WIF, followed by the interventions Outcomes, before describing how context affected both mechanisms and outcomes in the Context section. We first describe findings on the intervention's mechanism rather than initially focusing on the context to reflect the way in which WIF was conceived and presented to stakeholders, in 2013. WIF's design relied on the scarce body of evidence that the field had accumulated at that time, in addition to inputs from researchers and practitioners in the field. These inputs were used to determine the mechanisms that were deemed effective to prevent trafficking (the intervention's intended impact). Contextual variables were not part of these initial discussions and were also absent in the first iterations of the program's theory of change and log frame. The basic assumption that guided the development of WIF's community-based component was that women's empowerment would lead to reduced incidence of trafficking.

### Mechanisms of WIF

This section describes the main characteristics or mechanisms of the WIF intervention, i.e., what it was about the WIF interventions that brought about any effects in empowerment and information outcomes, and on the incidence of human trafficking in each intervention context. WIF's proposed mechanism was that predeparture training, targeting prospective migrants, would empower women, make them aware of their migration circumstances, thus changing their migration behavior, and ultimately protect them from human trafficking. We describe below the results from our research that relate to WIF's mechanisms, based on Dalkin et al. (37) conceptualization of mechanisms in realist evaluations (37). That is, we examine the mechanisms both as the resources offered by the intervention and the ways in which these resources change the reasoning of participants.

#### Intervention Content

In each country, the training was designed as premigration decision-making or premigration preparation sessions for prospective migrants, which aimed to help participants consider local employment opportunities, the pros and cons of migration, practical migration preparation, common problems encountered during migration, and emergency contacts. In Nepal, prospective migrants who completed the survey were followed by phone over time to track their migration planning and process. At the first follow-up interview, 50% of those reached reported having attended the WIF training (*n* = 94/188) and noted that the most important information they received was related to the documents required to migrate (62%) and legal travel routes (40%) (38). In India, while the training was well-received, with 95% of participants reporting that they learned information about migrating safely that they did not know before, assessments of participant learning indicated that all knowledge domains covered by the training remained low after the training (see *Outcomes* below) (30). In Bangladesh, although some women appreciated the content of the training, they often did not think the guidance was relevant to their lives, and some found certain information to be incorrect. As noted by one participant in Bangladesh who was informed of local livelihood options that did not materialize: “We can now tell anyone who wants to hear, that all these beautiful words about getting a loan are pure lies” (32).

There were also misperceptions about a “hotline” at destination in case of emergencies, which, for some, had serious implications, as in the case of one Bangladeshi woman who had migrated to a Gulf State:

Eleven days after arrival, a distressed Shikha [alias] called her husband and her mother and asks to be repatriated, who contacted the NGO fieldworker. She recommends that they let the phone keep ringing because Shikha is acting childishly and is not taking sufficient time to adjust. Shikha also calls the NGO helpline from the employer's home. The NGO social worker does not seem to understand the sexual abuse that Shikha cannot reveal (32).

While there was a “hotline” as promised, the support workers' ability to respond was not sufficient to meet this woman's actual needs. Similarly, in Nepal, women attending the training felt safer to migrate after having heard about contact details of the implementing partners because they believed they would be rescued if needed (39).

#### Target Participants

Participants for whom WIF intervention content was largely irrelevant were recruited across sites, which likely affected retention and interest in program messages. The WIF intervention's target participants were identified and recruited by local organizations commissioned by the WIF program. For example, in Nepal, peer educators conducted house-to-house visits to identify women interested in migrating, who would then be invited to the 2-day WIF training. This broad targeting approach, which treated all women of working age as potential migrants, was deliberate due to the stigma associated with women's labor migration and the need to achieve a maximum number of participants to meet donor obligations. Unsurprisingly, findings from Nepal suggest that many who were identified as “prospective migrants” did not actually have any clear plans to migrate but rather a loose idea of considering migration if the opportunity arises. Among the 188 women interviewed during follow-up surveys, only one-third (*n* = 58) stated that they still intended to migrate, while 15% (*n* = 27) had arrived at their destination. This somewhat loose participant selection process inevitably raised questions about the extent to which the participants were appropriately targeted and selected, which may also have had the knock-on effect of lowering women's uptake of the training information. Among 347 training participants in India, just 10.4% had thoughts of moving away from their village before the training, which also reflected low female outmigration patterns in which only 7% of households had a female migrant (30). As with Nepal participants, the low proportion of training participants considering migration likely indicates poor participant identification. Poor intervention targeting was also reflected by the low numbers of women who actually migrated for domestic work in the India study site (vs. construction and agriculture, which were not WIF's focused sectors).

Participants also seemed to have mixed motives for agreeing to attend the training sessions. For instance, several participants in Bangladesh indicated that they joined primarily for the free meal. Numerous participants also had misguided expectations of the “premigration training,” explaining that they thought the training would provide them direct assistance to migrate. As a Bangladeshi participant noted: “I went to the [NGO] training hoping I would get a visa and they would help me to migrate but got nothing… [the NGO] needed us… Now they are finished with us.” As she indicated, some perceived that their participation was more beneficial to the program than to themselves (32).

#### Implementation

##### Timing

Each 2-day training program was implemented on a specific date and time, which may have meant that participants were receiving the information too far in advance of their possible travel to retain the knowledge when they needed it. In Nepal and India, the training sessions focused on helping women decide whether or not to migrate, which meant some would only be deciding to migrate after the training. As noted by one Bangladeshi participant: “I liked everything about the training. The way the sister talked, the food…. But if you ask me what was said I could not tell you. I forgot most of it.” The migration planning process can happen very quickly or extend over a year or more, suggesting that “one-off” interventions may not meet actual needs as well as ongoing services and guidance. In India, training was very well-received, with 98.8% stating that they could follow along and understand the information given and 94.1% reporting that they would recommend the training to other women, but at the same time, there were extraordinarily low levels of actual learning (see *Outcomes* below).

##### Trainers

Observations about implementation also raised questions about whether the sessions were delivered by the right trainers. In a number of the sites, the training sessions were led by women who were of a higher socioeconomic class than the participants and who rarely if ever had their own migration experience. This social class difference may have led to participants' reluctance to accept some of the messages because of the messenger—especially messages pertaining to rights and empowerment. As one participant in Bangladesh explained: “I liked what they said about rights. There is nothing wrong with these beautiful words. But this kind of talk is not for us. It is good for educated people like you [pointing to the researcher]. What do we do with these nice words? We cannot implement them.” In Nepal, the training sessions were conducted by a peer educator from the implementing partner, alongside a social mobilizer who may or may not have had migration experience. In India, training sessions were conducted by implementing NGO's staff, and it was unclear whether any had migration experience.

### Outcomes of WIF

WIF's intended outcome was “Women are empowered to make informed migration decisions and an enabling environment is created for their safe migration into decent work.” The program's theory relied on the premise that this outcome would lead to the intended impact of “reduced incidence of trafficking of women and girls within and from India, Bangladesh and Nepal into domestic and garment sectors, through economic, social and legal empowerment.” This section describes the outcomes of the WIF premigration sessions for women, specifically addressing women's understanding and ability to acquire and apply a greater sense of *empowerment* and *information to find safe and decent work when migrating*.

#### Uptake of Empowerment and Rights Attitudes

The WIF training sessions sought to improve women's perception of the value of their work and their rights, both inside and out of the home. Among the 94 women in Nepal that attended the training, most appreciated messages about their rights, but only 2% indicated that the most important knowledge they gained was about the “rights of migrant workers” (of 20 items) (39). Low interest in worker rights may have been due, in part, to the fact that most women did not yet know the destination to which they would be migrating—or if they would be migrating at all. Actually, one in two Nepali women planning to migrate in the next 3 months knew nothing about contract details, including salary, living situation, hours, time off, contract length, or penalty for leaving early.

Interviews with women who had previously migrated indicated that migration itself conferred a sense of empowerment. Many Nepali women described feeling empowered via earning their own income, deciding how to spend their money, and their wider knowledge of the world from going abroad. Conversely, findings also suggest that the association of women's migration with promiscuity was stigmatizing for some returnee women who described being gossiped about when they returned (40). Moreover, in settings such as Nepal, where women's labor migration is a relatively recent phenomena, women who challenge traditional gender roles are frequently stigmatized, and these concerns were not addressed by the WIF intervention.

The erroneous assumptions hindered outcomes on empowerment. In India, women's attitudes toward women's work remained relatively fixed even after the training. For example, when asked about the statement, “Woman's work is not as important as men's work,” before the training, 47.3% agreed, which only changed to 44.9% afterwards. Attitudes toward domestic work also remained broadly negative, with 43.6% of participants agreeing that “Sita should feel ashamed to do paid domestic work in someone else's home,” before the training compared with 46.7% after. Despite these findings, small positive changes were observed for respect and rights. For example, before the training, 47.3% agreed that “Paid domestic workers have the same rights as all workers,” which increased to 59.9% after. After the training, a majority reported they learned about women's rights (85.6%) and workers' rights (84.9%), but these changes were not reflected in their reported attitudes about these concepts (30).

#### Uptake of Migration-Related Learning

Outcomes on migration-related learning were also relatively low. In India, women's pre- and post-knowledge of migration risks and opportunities was assessed using single questions connected to a migration-learning construct, such as, “What would you say are the main risks in moving away from home to take up work somewhere else?” Before the training, participants could cite an average of 1.2 risks (of 13) compared to 2.1 risks after the training. Similarly, low learning scores were observed for virtually all topics included in the training, indicating very low to zero retention of training messages (30).

In qualitative interviews in Bangladesh, women's perceptions of risk following the training did not reflect WIF messages. For example, advice from the WIF training to migrate without a *dalal* (informal labor intermediary) was strongly rejected (32). Women believed it was necessary to use a *dalal* to migrate. While women recognized numerous risks related to migration, many believed that outcomes were due to chance, and some believed that spiritual practices would protect them. Participating in WIF training was interpreted by some women as a kind of “certificate,” which lent them a special advantage that would reduce their risks during migration (32). However, at the same time, women did not often follow the advice they received in the training (e.g., travel via formal routes and agencies). In qualitative interviews in Nepal, one woman reported that she learned she should obtain necessary migration documents and travel via safe routes. Nevertheless, she traveled through an irregular channel following the advice of her broker and discussions with her husband. These types of decisions suggest that WIF messages may be constrained by individual and contextual realities, spiritual or superstitious beliefs, and structural forces.

#### Reduction in the Incidence of Human Trafficking

Findings from Nepal indicate the premise that women's empowerment and awareness prevents human trafficking is misguided in that context. Instead, our results indicate that the most important risk associated with forced labor is their country of destination, which is determined by the labor recruiter. Women's individual characteristics, awareness, and participation in trainings did not affect their likelihood of unfree recruitment, work and life under duress, or impossibility of leaving their employer (the three dimensions of forced labor, as defined by the ILO) (20). Data from Bangladesh suggest that this might also have been the case in that context, where qualitative accounts of posttraining migration show the difficulties women had in implementing WIF's empowerment strategies, as described above.

### Context

This section analyses WIF's outcomes in the context of the underpinning theory and assumptions. The contextual conditions needed for the intervention mechanisms to be activated (41) were not theorized previous to WIF's implementation. The results presented in this section describe how these contextual conditions interacted with WIF's theory.

#### Formal vs. Informal Recruitment Agents in Migration

A core assumption in the WIF theory of change was that migrant women should and could use formally registered recruitment agents (vs. informal brokers). Therefore, a central WIF program message discouraged the use of informal brokers in favor of registered agents and included guidance about how women could secure the required travel documents, negotiate formal work contracts, and travel via legal migration channels. Our findings indicate that, in reality, many, if not most, of the women planning international migration have to engage with informal agents at some point in their journey (42), especially women who came from rural areas where there are few or no registered agents. Rural women in Bangladesh had greater trust in local recruiters because they had the impression that recruiters would be accountable in some way if something went wrong—even if plans would eventually involve registered agencies (43). In India, women migrated almost exclusively within India. Only 13.9% of migrant women relied on a broker. The vast majority of women's migration was facilitated by their friends or acquaintances without participation of a labor contractor (72.2%). Among those who used brokers, deception was commonly reported: “I had no idea to find out my whereabouts. The person (broker) was maintaining tight secrecy for which we were in doubt. We started querying to the broker regarding our work settlement. I was not in a favor of working in anybody's house. The broker had told us to wait till the next morning. But, he had cheated us.”

#### The Influence of Rights and Empowerment Messages Amidst Local Gender Norms and Socioeconomic Power Dynamics

A further assumption in the WIF theory of change was that empowerment training could lead women to exert their rights as women and as workers. However, one influential barrier hindering this behavior change may have been women's very low confidence in their own ability to assert their power in their communities or within their families—particularly in the face of the pervasive gender inequalities, especially the cultural norms related to female migration. In India, over half of the returnee female migrants (53.5% of *n* = 112) reported they had limited input in the decision concerning their migration. In Bangladesh, women explained that they would have great difficulty expressing the empowerment messages they heard in the training. Exercising their rights became even more problematic when women needed to negotiate with labor brokers and nearly impossible with employers once they arrived in the destination, not least because work conditions are rarely negotiable and rights related to foreign workers are not enforced even if they exist on paper, particularly for domestic workers (44). In other words, knowing about their rights could not protect women if the context did not provide space to assert those rights. It is not a coincidence that forced labor among Nepali female returnees was higher in countries with the *kafala* system at the time of the study, which gave employers full state-sanctioned rights over migrant workers, including their visas (45).

In addition, while the WIF intervention tried to normalize migration for the WIF participants, female migration in South Asia is still highly stigmatized because of its association with sex work or promiscuity (40). Nearly half of female returnees in India (48.6% of *n* = 112) reported suffering stigma when they returned home. In this sense, in women's own community, WIF's messages were hampered by the social reality of women's lives, where entrenched social norms and accepted power structures pose substantial barriers along the WIF-anticipated pathways toward empowerment, particularly when empowerment is specifically related to migration.

A further underlying, albeit more distal, assumption was that if women could be equipped with correct knowledge about migration and assert their knowledge with recruiters (e.g., contracts, rights, migration regulations), this could influence recruiters to change their behavior. That is, if women knew how to negotiate their contracts and understood the laws and their rights and necessary documentation, recruiters would have to treat them differently, i.e., could not exploit them. Yet to date, there is little to no evidence from our study or elsewhere indicating that intermediaries alter their practices—exploitative or not—if women are more knowledgeable about the migration processes. Conversely, our study showed that having contracts did not protect Nepali migrant women from forced labor at destination (20).

#### Risk Awareness Among Target Populations vs. Risk Tolerance

Another underlying WIF assumption was that being aware of the risks of forced labor can make one safer from exploitation—an assumption that has underpinned many trafficking prevention activities around the world (6). Yet, findings from our research and other studies indicate that most prospective migrants are actually aware of migration-related risks. Nevertheless, individuals frequently maintain fervent hopes that migration will work out for *them personally*, or are willing to take their chances, even if they believe there are risks for *other people* (10, 46). Among SWIFT participants in Nepal, just over two-thirds (67.8%) of returnee migrant women reported having been aware that migrants may be deceived about their working terms and conditions prior to migrating. Most reported (91.2%) having suffered forced labor. Additionally, data from the Nepal survey with returnees and prospective migrants suggest that past experiences of forced labor are also not related to whether or not a respondent decides to return to the same destination or sector in which they were previously exploited. There does not even seem to be a “dose response effect,” as participation in trainings did not reduce the likelihood of experiencing forced labor—even among those women who had a work contract, which was suggested to be protective according to the WIF training (20).

#### Language Capability and Exploitation

Local language skills can influence women's negotiating power, but this was not a strong component in the trainings. For example, even for interstate migration in India, language barriers can affect their engagement at destination. As one study participant in India explained: “In Kerala, I could not understand their language. They too do not understand our language, I suppose. I felt like, I was mum almost all the time. There was only work, all the time. We could not take rest for a while during the duty hours. This is so exhausting. I feel very tired there. On the top of this, there is hardly any chance of social interaction. Sometimes, I feel like damning those people there” (29).

Among Nepali returnees who had migrated to Arabic-speaking countries, almost three in four (73.6%) reported that they could speak some Arabic. The assumption that they would be able to negotiate their contracts or working conditions in Arabic seems, however, farfetched. Results from regression analysis suggest that the prevalence of forced labor among returnees was not associated with speaking the language [crude odds ratio (OR), 0.75; 95% CI, 0.74: 4.0; AOR, 1.1; 95% CI, 0.41: 2.8]. Actually, the prevalence of forced labor was slightly higher among women who could speak the language (95.7%; 95% CI, 93.0: 97.6) vs. women who did not speak the language (92.8%; 95% CI, 86.8: 96.7).

#### Learning Uptake Among Participants

An important assumption in the WIF design was that the invited participants would be interested in, engage with, and take up the learning offered in the WIF curriculum. Yet, findings cast doubt on the learning uptake by participants. Although the vast majority of WIF training participants in Odisha reported previous training on worker's rights or labor migration, WIF participants' initial knowledge scores about migration risks, practices, rights, and collective bargaining were very limited, and their scores remained low even after the 2-day training, as noted above (30). Low learning levels might be explained by low prevalence of female migration (7% of households) in the site that ILO selected for the training and evaluation, which likely hindered participant interest and uptake of migration-related information. This finding was similar to the prevalence of female migration in the formative research site in Dolakha, Nepal (2%). As a result of the formative research, this site was dropped from the evaluation. Another possible reason for low learning uptake In India might have been the substantial training focus on domestic work when the larger portion of female migrants worked in construction (25.2%) vs. paid domestic work (19.1%). Moreover, women in India may not have been interested in the exploitation and trafficking messages offered in the training because of the generally low levels of reported forced labor (4%), especially when compared with Nepal (91.2%). At the same time, however, Indian women who had migrated within India working in non-domestic work sectors reported fairly poor work conditions, with 53% indicating that they worked in a dusty, smoky, fume-filled space without adequate ventilation; 50% did tasks that could hurt them or cause illness or sickness; and 37% were in uncomfortable or painful positions for long periods (47). Yet, occupational risks and protections were not part of the WIF training curriculum. Furthermore, in qualitative interviews, migrants in India reported non-payment of wages, reduced wages, or delayed payments, as well as restrictions in freedom, violence, and abuse by employers. One interviewee reported her experience: “In Kerala, the sister's (female employer) husband misbehaved with me. So at that time I was frightened. And I wanted to go home. About this matter I couldn't speak to anyone, because language was a big problem. Toward the end of my 5 years stay, I deliberately became more uncooperative…. The unpleasant experience of sexual assault by that old man in Kerala has indeed left a lifetime scar on me. I used to spread a mat under the bed of a room and sleep there with fear like a dog.” Despite regular evidence from this study and others, abuse, especially sexual abuse, was not integrated into the curriculum.

## Discussion

Intervention-focused evaluation on human trafficking, forced labor, extreme exploitation—or “modern slavery” —is a relatively nascent field of study. As human trafficking is an emerging area for interventions, it is not surprising that findings on trafficking prevention currently suggest more weaknesses than strengths in the WIF intervention theory, assumptions, mechanisms, and outcomes. Moving forward toward more effective and cost-effective programming, results from this evaluation offer crucial insights about future antitrafficking prevention programming that relies on premigration training. Our findings add to the growing body of evidence on the ineffectiveness of premigration knowledge building as an antitrafficking strategy (10, 15). Furthermore, this evaluation highlights the importance of understanding the full context in which interventions are intended to have an effect, not solely the site where the intervention is delivered. This study also has implications for future methodological approaches to intervention development and evaluation.

First and foremost, when addressing modern slavery, and even when considering “everyday exploitation,” there is no denying that the dynamics in each context play the most significant role. The asymmetric power relationship between workers and others involved in their recruitment and employment will continue to hinder mechanisms such as premigration training from achieving the outcome of making women safer because it relies on women to be able to apply these rights and information. In other words, while the WIF intervention's messages about women's rights as women and their rights as workers are undeniably inherently beneficial, it nonetheless remained unreasonable for women to believe in these rights and even more difficult for them to assert them in settings in which these rights are rarely, if ever, respected (48). Exerting these rights is especially difficult in countries where employment contracts bind employees to employers through migration sponsorship programs, such as the Kafala system in the Gulf countries. Evidence from our research and many other studies show that migrants face increased risks of exploitation, violence, and reduced access to justice under this system (20, 48–50).

**Figure 2:**
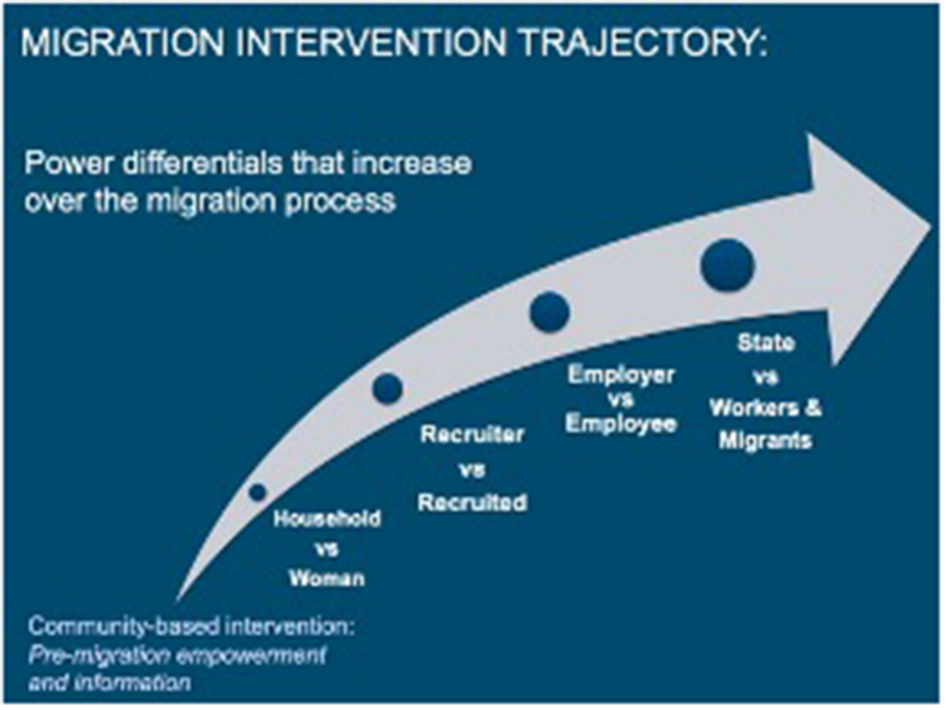
Migration intervention trajectory and increasing power asymmetries.

This context of often-extreme power imbalances suggests that it may be problematic to invest in premigration interventions alone, as these single location initiatives do not take sufficient account of the full migration trajectory, especially the later stages toward the destination and workplace, where the power asymmetry widens and most of the abuses occur. Because women themselves are not able to confront the entrenched and unequal power dynamics with recruiters or employers, interventions must be designed to address multiple points in a woman's migration trajectory. Figure 2 highlights how power asymmetries grow over the course of migration, which will limit the potential effectiveness of interventions that solely aim to arm women with knowledge before they migrate without addressing the increasing power differentials women encounter throughout their migration journey. The WIF community-based intervention's theoretical assumption, that if women were equipped with the migration-related knowledge, awareness of their rights and a budding sense of empowerment, they could manage the forthcoming negotiations, was not credible in the face of the severe inequalities related to gender, socioeconomic status, and migrant discrimination, particularly the state-sanctioned inequalities in destination locations. While there have been international legislative achievements that should influence local laws, such as the adoption of the International Labor Organization's Domestic Workers Convention 189 (51); to date, there have been only 29 ratifications, few of which include common destination locations for migrant domestic workers and no Gulf states. Moreover, while these tools lay the essential scaffolding for future improvements, there is little evidence of their earnest implementation and even less evidence that women workers experience any enforcement or benefit from regulatory provisions. These intervention challenges were also noted in the 2020 report by the United Kingdom's Independent Commission for Aid Impact (ICAI), “It also makes little sense to address international trafficking and forced migration in source countries without also taking necessary action in destination countries and along migration pathways” (52). Unfortunately, the original Work in Freedom programmatic theory of change for the community-based activities relied on assumptions about the potential for individual empowerment and awareness raising to translate into reduction in vulnerability when migrating. However, WIF did engage local implementing partners that worked closely with migrant populations or had expertise with gender issues.

While WIF recruited a broad range of participants, many anti-trafficking programmes target participants based on poverty, sex and education status as supposed “risk factors”, which are non-modifiable characteristics that generally fall beyond the scope of anti-trafficking programming. Findings from our work suggest that identifying at-risk groups based on these individual indicators is likely to be misleading at best and wasteful at worst. Moreover, this study indicates that individual situations have to be carefully considered in the gendered and socioeconomic context to avoid assumptions about the promise of empowerment against exploitation. That is, programs to prevent labor exploitation among international migrants need to address more than just the predeparture characteristics of prospective migrant women but must consider interventions that shift the power imbalances for migrant workers in relation to larger structural determinants, particularly recruitment processes and the employment context at the end of the migration trajectory. Targeting modifiable determinants of a migrant's experiences, for example, recruiter behaviors, which fall further up the structural pathway, may yield better results (see Figure 2).

Furthermore, the political context of interventions matters. Definitions of trafficking and forced labor are highly contentious, and governments of both origin and destination locations often dispute the use of these constructs and measurements to describe the situation of their citizens or their foreign workers. During the course of our evaluation, there was some resistance to the subject of forced labor and human trafficking. Moreover, the release of prevalence measurements of trafficking and forced labor was seen as particularly sensitive to governments and the international organizations operating in these sites. In particular, international agencies were concerned about the potential negative implications of the numbers of migrants being exploited and feared that the findings would cause government to (re)instate the female labor migration bans (53).

From a realist evaluation perspective (21), WIF lacked some clear initial theorizing about what were potentially successful pathways to influence change in deeply constrained migration trajectories. Moreover, having the programmatic ceiling of accountability fixed on women's empowerment and awareness meant that the actual effectiveness of the program was left mostly unchecked throughout implementation, with only the emerging findings from SWIFT, especially the qualitative component in Bangladesh, able to reveal some of the unintended and often harmful outcomes. Comprehensive monitoring systems that inform a program's adaptation would have been necessary to ensure prevention of harm via early detection of unintended outcomes.

### Limitations

Methods for data collection and analysis were not the same across sites. In each setting, we prioritized robust methodological approaches that were complementary and could address our main evaluation question, i.e., to what extent the rationale and assumptions of WIF were supported by context-specific evidence, and what were main enablers and barriers to implementation. We relied on the knowledge and experience of our local partners to ensure that we implemented feasible, acceptable, and ethical methodological approaches for research in each site. Triangulation across sites showed many similarities in findings, even considering the different methods used for data collection. This consistency in findings provided strong indications of the strength of the evidence produced by SWIFT on WIF's theory and implementation.

Of the four cross-sectional surveys we conducted, two relied on representative population samples. Unfortunately, both were conducted in WIF intervention sites with very low prevalence of female migration (Dolakha in Nepal, and Odisha in India). These sites were initially selected by the ILO to be the focus of the SWIFT evaluation, but Dolakha was ultimately removed for Nepal. The surveys with prospective and returnee migrants in three other Nepal districts relied on WIF's strategy for identification of their target population. This strategy aimed to avoid underreporting of migration experiences and aspiration by relying on identification of migrant women by peer educators hired by the ILO's local partners that operated in the specific districts. This population cannot, therefore, be considered representative of the overall population of female migrants living in the evaluation sites. We can, however, safely assume that they represent WIF's target population, since these were the women who were invited to participate in WIF's activities.

Loss to follow-up was very high in longitudinal data collection in India and Nepal, especially after women left home. We put in place several strategies to reduce attrition, but we were not able to conduct many interviews after women reached their destination. Findings on outcomes, in particular (especially quantitative data), were limited by the ethical and logistical challenges to follow women throughout their migration journeys, especially women in exploitative, abusive, or unfree work conditions. In Nepal, follow-up was particularly challenging, partly due to the 2015 earthquake that occurred at the end of the fieldwork. In the aftermath of the earthquake, women's contact details and migration plans may have changed, which may explain in part why there was such a large number lost to follow-up. Details of limitations in each of the individual studies are available elsewhere (20, 28, 30, 34, 35).

We acknowledge that the WIF intervention involved more than the community-level activities and also worked with governments, trade unions, employers, and others. These other components may have had positive effects, but our remit with SWIFT was to evaluate only the community-level activities.

## Conclusion

This theory-based evaluation strongly indicates that the community-based component of the WIF intervention was not sufficiently researched or well-designed to prevent the exploitation of migrant women. This evaluation also contributes further evidence against premigration knowledge-building and awareness-raising because they are extremely unlikely to sufficiently equip migrants to overcome the power inequalities that they will encounter throughout the migration journey, especially once they are in their employment situation. While these findings are disappointing, they are nonetheless crucial for the field of human trafficking prevention precisely *because* the results demonstrated that the intervention did *not* work. There is growing recognition of the necessity to publish null findings of rigorously produced evidence for interventions that do not do what was intended and offer explanations for apparent failure. Scientists reporting weak or null findings are arguably among the most valuable soothsayers in policy or programming realms because of their ability to explain what should be done differently or more effectively in the future, to avoid the waste of precious funds (54). While our research did not indicate that the intervention was effective in reducing women's risk of exploitation, the results most certainly emphasized that women who decide to migrate for work need and deserve better migration policy protections and effective interventions, so they can be safe when they strive for better livelihoods for themselves and their family.

## Data Availability Statement

The original contributions presented in the study are included in the article/supplementary material, further inquiries can be directed to the corresponding author/s.

## Ethics Statement

The studies involving human participants were reviewed and approved by Ethics approvals were obtained from the London School of Hygiene and Tropical Medicine (LSHTM) (reference numbers 8840, 7021, and 8895), the Centre for Women's Development Studies, Indian Council of Social Science Research for India, the Nepal Health Research Council (reference numbers 1040 and 1441) and the South Asian Network on Economic Modeling, an autonomous research institute in Bangladesh. The patients/participants provided their written informed consent to participate in this study.

## Author Contributions

CZ and LK: conceptualization, funding acquisition, supervision, and writing—original draft. LK, JM, and NP: data curation and formal analysis. CZ, LK, JM, and NP: investigation, methodology, and writing—review & editing. All authors contributed to the article and approved the submitted version.

## Conflict of Interest

The authors declare that the research was conducted in the absence of any commercial or financial relationships that could be construed as a potential conflict of interest.

## Footnotes

^1^“Ceiling of accountability” is the distinction between factors amenable to change from the intervention compared to factors that cannot be controlled by the intervention (beyond its scope) (26).
