# Corrigendum: Human trafficking: Results of a 5-year theory-based evaluation of interventions to prevent trafficking of women from South Asia

**DOI:** 10.3389/fpubh.2023.901443

**Published:** 2023-02-09

**Authors:** Cathy Zimmerman, Joelle Mak, Nicola S. Pocock, Ligia Kiss

**Affiliations:** ^1^Gender Violence & Health Centre, Department of Global Health & Development, London School of Hygiene & Tropical Medicine, London, United Kingdom; ^2^Lumos Foundation, London, United Kingdom; ^3^Faculty of Population Health Science, Institute for Global Health, University College London, London, United Kingdom

**Keywords:** human trafficking, modern slavery, migrant women, realist evaluation, South Asia

In the published article, there was an error in which a quotation was erroneously included.

A correction has been made to the **Conclusion** section, page 10. The sentence that has been corrected previously stated:

“Sadly, in the case of this large 5-year evaluation investment, the results did not appear to steer future investments that were made in the second phase of the WIF intervention. As noted by the Independent Commission for Aid Impact, “There is also no evidence that these findings were used to inform the design of the second phase of the work.””

This sentence has now been deleted.

The authors apologize for this error and state that this does not change the scientific conclusions of the article in any way. The original article has been updated.

